# The mediating effect of pregnancy adaptation between family support and maternal-fetal bonding: a cross-sectional study

**DOI:** 10.1186/s12912-024-02009-5

**Published:** 2024-05-24

**Authors:** Wan-Ru Wu, Li-Chun Lee, Chich-Hsiu Hung, Hui-Chuan Huang

**Affiliations:** 1 Department of Nursing, College of Medicine, Tzu Chi University, Hualien, Taiwan; 2 Department of Nursing, Asia University, Taichung, Taiwan; 3 School of Nursing, Taipei Medical University, Taipei, Taiwan

**Keywords:** Family support, Pregnancy adaptation, First trimester of pregnancy, Maternal-fetal bonding

## Abstract

**Background:**

Establishing a nurturing bond with the unborn child is essential for expectant mothers throughout pregnancy. While the influence of family support and pregnancy adaptation on maternal-fetal bonding is evident, these factors remain unexplored in the early stages of pregnancy. This study aims to elucidate the dynamic interaction between family support, pregnancy adaptation, and maternal-fetal bonding during the first trimester, explicitly investigating the mediating role of pregnancy adaptation.

**Method:**

A cross-sectional design was conducted to recruit expectant mothers between 8 and 12 weeks of gestation without significant complications.

**Results:**

Family support and pregnancy adaptation emerged as significant predictors of maternal-fetal bonding, and pregnancy adaptation mediated the relationship between family support and maternal-fetal bonding in the first trimester.

**Conclusions:**

The study confirms the critical role of family support and pregnancy adaptation in facilitating maternal-fetal bonding during early pregnancy, with pregnancy adaptation fully mediating this relationship. Healthcare providers are encouraged to involve family members in early interventions, focusing on assessing family support and engaging them in education and activities to strengthen the emotional bond between the mother and her unborn child.

## Background

The pregnancy journey signifies a profound and challenging period for expectant parents. Maternal-fetal bonding (MFB) begins early in pregnancy as the mother becomes aware of her condition, laying a vital foundation for secure attachment crucial for the infant’s social-emotional development [1, 2]. MFB entails affectionate feelings, a sense of protection, the establishment of interaction, and an emotional connection between the mother and her unborn child [3]. Research suggests that MFB levels increase progressively throughout gestation, with lower levels typically observed during the first trimester [4, 5]. However, despite its significance, interventions aimed at enhancing MFB, such as responding to fetal movements and engaging in prenatal activities, are often concentrated on the later stages of pregnancy, neglecting the critical period of the first trimester [6–8].

Throughout the challenges of early pregnancy, the presence of family support emerges as a significant factor influencing MFB [3]. Studies indicate that expectant mothers lacking a partner or family support during pregnancy often exhibit lower levels of bonding with their fetuses [2, 4]. Qualitative interviews with expectant mothers during the first trimester experiences reveal ambivalent emotions oscillating between happiness and worry, especially concerning miscarriage or fetal health issues. This uncertainty holds back their inclination to bond too early. It hinders their smooth transition into motherhood, as they may only feel comfortable expressing their emotions with close family members and partners [9, 10]. Consequently, robust support from family members acts as a protective buffer, fostering the development of MFB during this crucial period.

While family support is crucial for expectant mothers’ bonding development, assisting expectant mothers’ pregnancy adaptation has been evident to increase their levels of MFB. Although the exact definition of adapting to pregnancy varies, it typically involves multidimensional practices, including navigating physical and psychological changes experienced throughout gestation and adapting to the role tasks accompanied by pregnancy [1, 11, 12]. Previous research conducted with quasi-experimental designs using group training forms or personal consultation approaches found effective improvement in MFB [11, 13]. These programs often include embracing the pregnancy experience, managing physical changes, and maintaining a healthy pregnancy by monitoring weight gain, nutrition, and prenatal checkup outcomes. However, these clinical education programs often prioritize interventions during later stages of gestation to improve these outcomes.

**Fig. 1:**
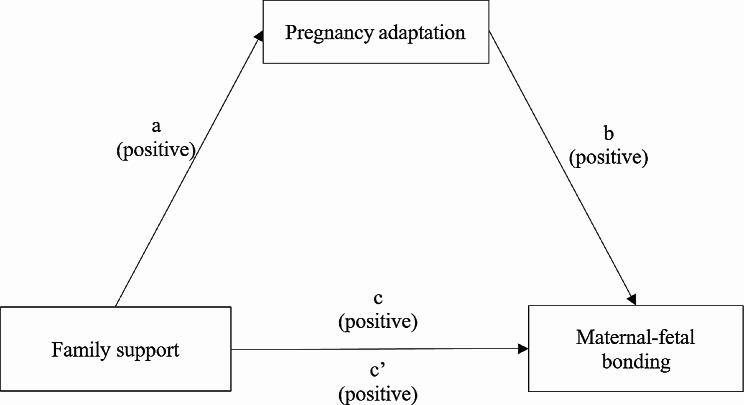
Conceptual model of the mediating effect of pregnancy adaption on the relationship of family support and maternal-fetal bonding. **a** Effect of family support on pregnancy adaptation; **b** Effect of pregnancy adaptation on maternal-fetal bonding; **c** Total effect of family support and pregnancy adaptation on maternal-fetal bonding; c’ Effect of family support on maternal-fetal bonding after pregnancy adaptation was added to the model

Despite the significant uncertainty experienced during the first trimester, the interplay between family support, pregnancy adaptation, and maternal-fetal bonding (MFB) remains underexplored. Previous evidence suggests that family support and pregnancy adaptation influence MFB. However, to our knowledge, the potential mediating relationship between family support and MFB through pregnancy adaptation in the first trimester has not been thoroughly investigated in previous studies. Building on the current literature, this study hypothesizes that family support enhances expectant mothers’ pregnancy adaptation, thereby positively impacting MFB, as shown in Fig. 1. Specifically, we propose that pregnancy adaptation may mediate the relationship between family support and MFB. Therefore, the study aims to investigate these hypothesized relationships and explore the potential mediating role of pregnancy adaptation during the first trimester.

## Methods

### Study design and setting

A cross-sectional study was conducted from December 2018 to September 2019 after receiving approval from the Institutional Review Board. Convenient sampling was employed at two obstetrical outpatient clinics in a rural private general hospital in northern Taiwan. In Taiwan, prenatal care is provided through the tax-funded National Health Insurance (NHI) system, which includes ten essential checkups for all expectant mothers confirmed by a licensed obstetrician. Upon confirmation of pregnancy, women are provided with an official Maternal Health Education Handbook, which documents essential data such as urine protein/sugar, blood pressure, and body weight at every prenatal checkup. Most expectant mothers choose their trusted obstetrician near their home and give birth at the same hospital. They are required to bring this handbook to each prenatal checkup starting from the first confirmation of pregnancy.

### Participants and procedure

The study targeted low-risk expectant mothers without significant complications, such as vaginal bleeding or severe heart disease, requiring close monitoring at the time of data collection. Eligible participants were expectant mothers aged 20 years and above, between 8 and 12 weeks of gestational age. Those unable to read Traditional Chinese were excluded. A trained research assistant identified suitable candidates during routine checkups in the outpatient waiting area. She approached potential participants, provided a standard script explaining the study details, and screened for eligibility using their Maternal handbook. After obtaining paper-based informed consent, the research assistant assisted participants in completing self-reported questionnaires.

### Sample size

Sample size estimation was performed using G*power 3.1 software, indicating a minimum requirement of 110 participants based on an anticipated effect size of 0.15 [14], an alpha of 0.05, 80% power, and two predictive variables. The study ensured comprehensive reporting of its observational design by adhering to the Strengthening the Reporting of Observational Studies in Epidemiology (STROBE) reporting guideline [15].

### Instruments

#### Demographic data sheet

The demographic data collected encompassed age, occupation, education, living arrangements (with or without in-laws/relatives), family income, parity, and whether the current pregnancy was planned or unplanned.

#### Family support

A 5-item Family APGAR questionnaire [16] was used to measure family support. The APGAR stands for five constructs: Adaptability, Partnership, Growth, Affection, and Resolve. Examples of items on the scale include “I am satisfied that I can turn to my family for help when something is troubling me. Responses to the scale are rated on a five-point Likert ranging from 1 (“never”) to 5 (“always”), with a total score of 25 calculated by summing the individual item scores. Higher scores indicate a higher frequency of family support perceived in early pregnancy. The scale has shown reasonable validity and reliability [17, 18], and the Cronbach alpha was 0.95 in the current study.

#### Maternal-fetal bonding

A 39-item Modified Maternal-Fetal Attachment Scale [19] was used to measure maternal-fetal bonding. The questionnaire contains emotional and behavioral attachment questions that were initially merged from Cranley’s Maternal-Fetal Attachment scale [20] and Muller’s Prenatal Attachment Inventory scale [21]. Examples of items on the scale include “I am curious about what the baby looks like now” and “I use a (the) nickname to call my baby.” Responses to the scale are made on a five-point Likert range from 1 (“never”) to 5 (“always”) and are summed to a total score ranging from 39 to 195. Higher scores indicate stronger Maternal-Fetal bonding. Several studies have shown good validity and reliability [19, 22], and the Cronbach alpha was 0.94 in the current study.

#### Pregnancy adaptation

A 28-item pregnancy adaptation scale [23] was used to measure maternal adaptation to pregnancy. Examples of items include “I am satisfied with the role of the pregnant woman,” “I accept the changes coming along with the pregnancy,” and “I care about good nutrition during my pregnancy.” Responses to the scale are made on a four-point Likert from 0 (“never”) to 3 (“always”) and are summed to a total score ranging from 0 to 84. Higher scores on the scale indicate better pregnancy adaptation, as they reflect a greater level of satisfaction with the role of expectant mothers, acceptance of the changes that come with pregnancy, and concern for good nutrition. The scale has shown acceptable validity and reliability [23, 24], and the Cronbach alpha was 0.92 in the current study.

### Statistical analysis

The data were analyzed using the SPSS 20.0 software program (IBM. Corp., Armonk, NY, USA). Descriptive statistics were used to assess participants’ characteristics. Differences in main variables based on participants’ characteristics were examined using ANOVA with post hoc tests, and simple correlation analyses were applied to explore bivariate relationships and identify significant association. Subsequently, correlation analysis was conducted to explore the associations between family support, pregnancy adaptation, and MFB.

To examine the proposed hypothesis model (Fig. 1), family support was treated as the independent variable, pregnancy adaptation as the mediator, and MFB as the dependent variable. Three hierarchical linear regression models were employed: Model 1 assessed whether family support significantly predicted MFB, Model 2 tested whether family support significantly predicted pregnancy adaptation, and Model 3 examined whether pregnancy adaptation significantly predicted MFB while accounting for the influence of family support. Pregnancy adaptation was considered a mediator if the effect of family support on MFB was diminished or no longer significant in Model 3. Collinearity among demographic variables, family support, MFB, and pregnancy adaptation was assessed using variance inflation factor (VIF), with a VIF value of less than 10 indicating no collinearity.

The Process analysis proposed by Hayes [25] was utilized to confirm the mediating effect of pregnancy adaptation. This method computed the product-of-coefficient and explored the direct and indirect pathways through which an independent variable (X) transmitted its effect on a dependent variable (Y) via an intermediary (M). A statistically significant indirect effect and a substantial mediated percentage indicated the importance of the mediator. The indirect effect test adjusted for common covariates of the independent variable and mediator simultaneously, employing a bootstrapping method to obtain an accurate indirect effect coefficient for small sample sizes [25].

## Results

### Participant characteristics

Out of 150 expectant mothers recruited, 138 completed the questionnaires for analysis, with nine participants excluded for not meeting the inclusion criteria. Table 1 presents the participant characteristics. The average age of the participants was 32.62 years (*SD* = 3.95). Most participants were employed full-time (70.3%) and held an undergraduate degree (69.6%). The majority of couples were living independently (60.9%), and 56.5% reported a monthly family income between 40,000 and 80,000 NTD (1,333-2,666 USD). Approximately 55.8% were expecting their first child, with a similar percentage of planned (42.8%) and unplanned (42.0%) pregnancies.

**Table 1:** Participant characteristics and variations in main variables based on participant characteristics (*N* = 138)

Variable	All		FS M (SD)	PA M (SD)	MFB M (SD)
	*n*	%			
All participants			19.8 (3.9)	61.1 (12.6)	119.5 (26.3)
Age (years) (M, SD)	32.62	3.95	*t* = -0.10	*t* = 0.99	*t* = 0.06
Occupation			*F* = 0.44	*F* = 0.18	*F* = 0.20
Full-time	97	70.3	20.1 (3.7)	61.1 (11.5)	119.3 (25.8)
Housewife	27	19.6	19.6 (4.1)	61.7 (14.9)	121.7 (24.8)
Other	14	10.1	19.1 (5.1)	59.2 (15.9)	116.2 (33.2)
Education			*F* = 0.07	*F* = 0.37	*F* = 0.63
≤ High school	18	13.0	20.2 (3.6)	63.3 (14.7)	118.5 (24.8)
Undergraduate degree	96	69.6	19.8 (4.1)	60.6 (12.1)	118.3 (25.9)
Postgraduate degree	24	17.4	19.8 (3.7)	61.4 (13.4)	125.0 (29.1)
Living arrangements			*F* = 0.63	*F* = 0.72	*F* = 2.15
without in-laws	84	60.9	19.7 (3.9)	60.3 (12.5)	118.1 (23.1)
with in-laws	44	31.9	20.1 (4.1)	62.7 (11.8)	124.9 (28.3)
with other relatives/others	10	7.2	21.2 (3.5)	58.5 (16.7)	107.5 (38.2)
Family income ^a^ (NT$ per month)			*F* = 1.17	*F* = 0.66	*F* = 2.43
≤ 40,000	10	7.2	20.2 (5.0)	60.4 (16.2)	124.6 (27.9)
40,001 ∼ 60,000	34	24.6	19.1 (4.2)	60.7 (13.3)	122.6 (24.1)
60,001 ∼ 80,000	44	31.9	20.6 (4.1)	61.3 (11.0)	118.6 (25.2)
80,001 ∼ 100,000	23	16.7	18.9 (3.0)	57.1 (12.1)	105.7 (27.2)
≥ 100,001	27	19.6	20.3 (3.7)	63.9 (12.9)	126.8 (26.8)
Parity			*F* = 3.07	*F* = 3.96	*F* = 0.48
Nulliparous	77	55.8	20.4 (3.8)	59.2 (12.3)	118.1 (25.2)
Multipara	61	44.2	19.2 (4.1)	63.4 (12.7)	121.2 (27.7)
This pregnancy was			*F* = 0.04	*F* = 0.58	*F* = 0.19
Planned	59	42.8	20.0 (3.9)	62.4 (10.8)	120.6 (27.3)
Unplanned	58	42.0	19.8 (4.0)	60.1 (14.1)	117.8 (26.1)
Going with the flow	21	15.2	19.7 (4.1)	60.1 (13.2)	120.7 (25.2)

^a^ NT$ = New Taiwan Dollar; US$1 ≈ NT$30; EU$1E ≈ NT$35; FM = family support, PA = pregnancy adaptation, MFB = maternal-fetal bonding

^**^p < 0.01, ^***^p < 0.001

### Variations in main variables based on participant characteristics

The mean scores of all participants for family support, pregnancy adaptation, and MFB were 19.8 (*SD* = 3.9), 61.1 (*SD* = 12.6), and 119.5 (*SD* = 26.3), respectively (Table 1). No significant variations were observed in family support, pregnancy adaptation, and MFB scores based on participants’ demographic characteristics.

### Correlations among family support, pregnancy adaptation, and maternal-fetal bonding

Significant positive correlations were observed among family support, pregnancy adaptation, and MFB (Table 2). Specifically, family support had a positive correlation with both pregnancy adaptation (*r* = 0.44, *p* < 0.001) and MFB (*r* = 0.29, *p* < 0.001). Additionally, pregnancy adaptation had a positive correlation with MFB (*r* = 0.54, *p* < 0.001). The findings suggest that expecting mothers in the first trimester who perceive higher levels of family support are likely to have better pregnancy adaptation and stronger maternal-fetal bonding.

**Table 2:** Correlations among family support, pregnancy adaptation, and maternal-fetal bonding (*N* = 138)

Variable	FS	PA	MFB
Family support (FS)	1.00	**-**	-
Pregnancy adaptation (PA)	0.44^***^	1.00	**-**
Maternal-fetal bonding (MFB)	0.29^***^	0.54^***^	1.00

^***^p < 0.001

### Mediating effects of pregnancy adaptation

The mediating effects of pregnancy adaptation are presented in Table 3. A series of regression models were conducted to investigate the impact of pregnancy adaptation on the relationships between family support and MFB. In model 1, family support significantly affected MFB (*β* = 0.29, *p* = 0.001). In model 2, family support significantly impacted pregnancy adaptation (*β* = 0.44, *p* < 0.001). In model 3, with family support and pregnancy adaptation as predictors and MFB as the dependent variable, only pregnancy adaptation had a significant effect on MFB (*β* = 0.52, *p* < 0.001). Compared to the first step model, the effect of family support was reduced from significant to non-significant (*β* = 0.06, *p* = 0.43), suggesting that the effect of family support on MFB was entirely manifested by pregnancy adaptation. The VIF values in all three models were less than 1.3, indicating no multicollinearity.

**Table 3:** The effect of pregnancy adaptation on the relationship of family support and maternal-fetal bonding (*N* = 138)

Model	Predictor(s) on dependent variable	B	Adj *R*^2^	F
1	FS→MFB	0.29^**^	0.08	12.32^**^
2	FS→PA	0.44^***^	0.18	31.96^***^
3	FS, PA→MFB		0.29	28.80^***^
	FS→MFB	0.06		
	PA→MFB	0.52^***^		

^**^p < 0.01, ^***^p < 0.001 FS = family support, MFB = maternal-fetal bonding, PA = pregnancy adaptation;

**Fig. 2:**
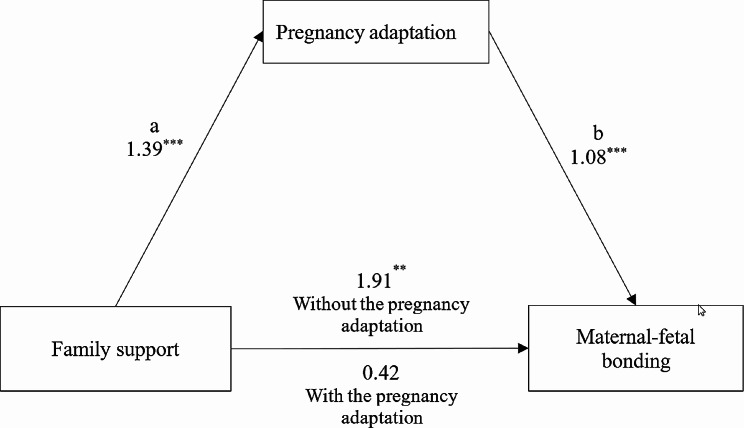
The indirect effect of Pregnancy adaptation is 1.49 (95% CI 0.91 to 2.13), and the percentage of mediation is 78.01%. **a**: The effect of family support on pregnancy adaptation is 1.39; **b**: The effect of pregnancy adaptation on MFB is 1.08; **c**: The effect of family support on MFB is 1.91 and decreases to 0.42 and is no longer significant after pregnancy adaptation is added to the model

As depicted in Fig. 2, the regression coefficients between family support and pregnancy adaptation, and between pregnancy adaptation and MFB were statistically significant. The total score for the indirect effect of the pregnancy adaptation was 1.49, with a 95% confidence interval ranging from 0.91 to 2.13. The confidence interval did not include zero, indicating that the indirect effect was statistically significant. The percentage of mediation was 78.01%. These findings suggest that most expectant mothers in their early pregnancy are more likely to have better MFB if their families provide supportive behaviors that promote pregnancy adaptation.

## Discussion

The study fills gaps in the existing literature by examining the interplay between family support, pregnancy adaptation, and MFB during the first trimester. Through investigating the mediating role of pregnancy adaptation, our findings provide novel insights into the mechanisms underlying the formation of MFB in early pregnancy. Our results validate the proposed model, illustrating how family support significantly influences expectant mothers’ levels of pregnancy adaptation, thereby shaping their capacity to form a strong bond with their unborn child.

Our findings resonate with existing literature revealing the emotional experiences of expectant mothers within their familial relationships [9, 10]. In many Asian countries, family serves as a primary source of support, and traditional cultural practices foster feelings of security and belonging among expectant mothers [26]. Even as Taiwanese couples increasingly trend toward living independently, they continue to receive extensive support from both sets of parents throughout the prenatal and postnatal phases, encompassing emotional care and practical assistance [27].

Additionally, our study highlights a significant challenge faced by current educational and parenting programs. These programs typically initiate during the second trimester, potentially overlooking the immediate needs of expectant parents during early pregnancy. Activities such as responding to fetal movements or listening to fetal heartbeat sounds [6–8] become impractical during this period, as the fetus is not yet palpable and the expectant mother’s abdomen remains flat. To bridge this gap, we recommend gradually introducing or training expectant parents in activities such as reading, talking, and singing to the baby during early pregnancy. These practices foster emotional and behavioral coherence within familial relationships and cater to the unique needs of expectant parents during this crucial period.

Moreover, it is crucial to acknowledge the information needs of expectant mothers, as highlighted in previous research [9]. Many express that pregnancy is profoundly real in their lives, and they imbue it with meaning through practices like information seeking, listening to their bodies, and anticipating pregnancy milestones. However, research on expectant fathers reveals feelings of exclusion at the beginning of the pregnancy [28], underlining the necessity for greater involvement in prenatal care. As family support often originates from partners, their inclusion in prenatal care is essential to ensure comprehensive support for expected mothers.

Further, our study investigated the role of pregnancy adaptation as a mediator, uncovering a significant indirect effect. This underscores the critical role of pregnancy adaptation and provides actionable insights into how family support can strengthen an expectant mother’s emotional connection to the fetus. Pregnancy adaptation, as measured in our study, encompasses dimensions such as embracing pregnancy, coping with physical changes, and engaging in healthy pregnancy practices like monitoring weight gain, nutrition, and attending prenatal checkups. Our findings align with previous research, which has shown that interventions focusing on pregnancy adaptation practices can effectively enhance MFB [11, 13]. While MFB has been extensively studied, our research introduces novel findings, suggesting that the early stages of pregnancy can be transformative for expectant mothers as they embrace pregnancy as a journey toward motherhood.

### Limitations

While this study contributes to the literature on MFB by investigating the relationship between family support, pregnancy adaptation, and MFB, potential limitations should be acknowledged. Firstly, the absence of variables such as prenatal depression and marital intimacy in the analysis could have influenced the observed relationships. Secondly, the study’s reliance on a cross-sectional design and recruitment of healthy expectant mothers from a single hospital may restrict the generalizability of the findings, particularly for high-risk pregnant populations.

Future research could employ longitudinal studies to examine the dynamic changes in family support and pregnancy adaptation on MFB across different trimesters to address these limitations. Moreover, diversifying participant recruitment to include individuals from various cultural backgrounds and contexts could offer a more comprehensive understanding of the mechanisms underlying the variables among expectant mothers during the first trimester. Such studies could help to overcome the identified limitations and provide a more comprehensive understanding of MFB.

### Implications for clinical practice

Healthcare providers should engage expectant couples early on, assessing family support and promoting activities to strengthen the emotional bond between the mother and her unborn child. Activities such as reading, talking, and singing to the baby establish bonding habits early on and help parents become more attuned to the changes of pregnancy. Additionally, involving partners and significant family members in prenatal care, along with practices promoting pregnancy adaptation, will facilitate meaningful bonding experiences and support expectant mothers by creating a supportive environment.

## Conclusions

Our study confirms the predictive influence of family support and pregnancy adaptation on MFB during the first trimester, with pregnancy adaptation mediating the relationship between the two. The findings highlight the importance of encouraging partners and family members to offer supportive practices early on in order to facilitate expectant mothers’ ability to adapt to pregnancy and develop bonding in the early stages of pregnancy.

## Data Availability

The datasets used and/or analyzed during the current study are available from the corresponding author upon reasonable request.

## Abbreviations

MFB: Maternal-fetal bonding
